# Assessing the relationships between phylogenetic and functional singularities in sharks (Chondrichthyes)

**DOI:** 10.1002/ece3.2871

**Published:** 2017-07-04

**Authors:** Marie Cachera, François Le Loc'h

**Affiliations:** ^1^ SHOM Brest Cedex 2 France; ^2^ UMR LEMAR CNRS/UBO/IRD/Ifremer, IUEM, Rue Dumont d'Urville, Technopôle Brest Iroise Plouzané France

**Keywords:** adaptive radiation, conservation, endangered clade, evolutionary convergence, niche conservatism, phylogenetic signal

## Abstract

The relationships between diversity and ecosystem functioning have become a major focus of science. A crucial issue is to estimate functional diversity, as it is intended to impact ecosystem dynamics and stability. However, depending on the ecosystem, it may be challenging or even impossible to directly measure ecological functions and thus functional diversity. Phylogenetic diversity was recently under consideration as a proxy for functional diversity. Phylogenetic diversity is indeed supposed to match functional diversity if functions are conservative traits along evolution. However, in case of adaptive radiation and/or evolutive convergence, a mismatch may appear between species phylogenetic and functional singularities. Using highly threatened taxa, sharks, this study aimed to explore the relationships between phylogenetic and functional diversities and singularities. Different statistical computations were used in order to test both methodological issue (phylogenetic reconstruction) and overall a theoretical questioning: the predictive power of phylogeny for function diversity. Despite these several methodological approaches, a mismatch between phylogeny and function was highlighted. This mismatch revealed that (i) functions are apparently nonconservative in shark species, and (ii) phylogenetic singularity is not a proxy for functional singularity. Functions appeared to be not conservative along the evolution of sharks, raising the conservational challenge to identify and protect both phylogenetic and functional singular species. Facing the current rate of species loss, it is indeed of major importance to target phylogenetically singular species to protect genetic diversity and also functionally singular species in order to maintain particular functions within ecosystem.

## 1 INTRODUCTION

The importance of biodiversity for ecosystem functioning becomes central in ecology (Cadotte, Carscadden, & Mirotchnick, 2011; Flynn, Mirotchnick, Jain, Palmer, & Naeem, 2011; Hooper et al., 2002; Naeem, Loreau, & Inchausti, 2002; Narwani, Matthews, Fox, & Venail, 2015; Srivastava et al., 2012). It is now recognized that taxonomic diversity is not the only component of biodiversity to measure, but instead that scientists have to focus on functional diversity as it is supposed to be directly linked to ecosystem functioning (Cadotte et al., 2011; Cumming & Child, 2009; Devictor et al., 2010; Hooper et al., 2005; Tilman, 2001). The main challenge is thus to correctly appreciate functions of species in order to estimate functional diversity. However, species functions are still complex to measure directly in the field, as we do not know neither all functions a species sustains in its ecosystem, nor their direct and indirect effects on ecosystem (Cadotte, Cavender‐Bares, Tilman, & Oakley, 2009). The first difficulty is to identify functional traits of species (Hooper et al., 2002; Mouillot, Graham, Villéger, Mason, & Bellwood, 2013; Petchey and Gaston, 2006); here, functional traits rely on functional effect traits following Srivastava et al. (2012). These functional traits should indeed be judiciously selected because (i) they need to be clearly identified as related to one or more functions of the ecosystem, and furthermore, (ii) they need to be quantitatively or qualitatively measurable. Now, within the field of functional ecology, as functional traits still appeared hard to define and measure, there is a growing interest on which proxy would be efficient to estimate the functional identity of species (i.e., its “role” within their ecosystems). A relevant proxy should ideally be relatively easy to measure and integrate several functions. Consequently, more and more authors started to focus on the representativeness of phylogenetic diversity for functional diversity (Cadotte, Albert, & Walker, 2013; Cadotte et al., 2009; Flynn et al., 2011; Guilhaumon et al., 2014; Mouquet et al., 2012; Prinzing et al., 2008).

Phylogeny can be estimated nowadays notably thanks to the development of barcoding techniques, its sharing and access through GenBank, and computational progress. As a consequence, if it allows to functionally identify a species, it may become a powerful tool to estimate functional diversity. However, the relationships between phylogeny and functions are still debated. On the one hand, a positive relationship detected between phylogeny and functions would imply that phylogenetically close species tend to be more similar in their traits (generally phenotypic) because traits were conserved along evolution. This hypothesis is supported by the principle of niche conservatism (Losos, 2008; Münkemüller, Boucher, Thuiller, & Lavergne, 2015; Wiens et al., 2010). On the other hand, it may be possible that not all species traits show conservatism, and thus that the relationships between phylogeny and functional identity would be weak or absent. For example, in the case of an adaptive radiation, species may have quickly diverged to avoid competition, and thus may be functionally separated despite their phylogenetic closeness (e.g., Darwin finches (Darwin, 1859; Schluter, 2000, 1996). On the opposite example, species may stay functionally closer than expected by their phylogenetic distances if traits are under a strong selection (Devictor et al., 2010). Under these last two theories, no or negative relationship between phylogenetic and functional diversities is expected. Facing all these contrasted contexts, it may be interesting to adopt a “clade‐based” point of view to study the relationships between phylogeny and functions. Using a single clade would indeed allow to focus on a single evolutionary context, as the relationships between phylogeny and function appeared to depend on the evolutionary history of the focal species (Srivastava et al., 2012).

Finding a relevant proxy for functional identity is a challenge that has to be quickly fixed in facing the current rate of loss of biodiversity. Conservation targets have to be wisely and rapidly chosen (Cadotte et al., 2013; Cadotte & Jonathan Davies, 2010; Díaz & Cabido, 2001; Petchey and Gaston, 2006, 2002a). Biodiversity is increasingly eroding and conservation planners need solutions to maximize their funds by targeting taxa or species which are key to ecosystem functioning and services. It is indeed generally admitted that species are not all equivalent, and that some of them may even be considered as singular, meaning that they are unique in the ecosystem. Their loss would thus not be compensated by another species (Cadotte & Jonathan Davies, 2010; Guilhaumon et al., 2014; Mace, Gittleman, & Purvis, 2003). Singularity is used here as a synonym of originality, a measurable rarity of a species' features, following Pavoine et al. (2005). These singular species, precisely because of their rarities, can be considered as the subset of species that should receive priority protection, particularly in the context of an acceleration of species loss (the Noah's Ark problem, Cadotte & Jonathan Davies, 2010; Devictor et al., 2010; Isaac, Turvey, Collen, Waterman, & Baillie, 2007; Weitzman, 1998). Phylogenetically speaking, singular species should be conservation priorities because their loss would imply a loss of genetic diversity, a key for species adaptation (Isaac et al., 2007). It is indeed of crucial importance to maximize genotypic diversity in order to allow biological systems to respond to futures changes in the world (Cadotte & Jonathan Davies, 2010). Functionally speaking, singular species should also have conservation priorities, because their functions would not be compensated by another species' functions if they go extinct (“insurance hypothesis”, Yachi and Loreau, 1999), and thus may imply a direct impact on ecosystem functioning (Hooper et al., 2005; Loreau, Naeem, & Inchausti, 2002; Mouillot et al., 2013). This impact would be even stronger if these singular species are in fact keystone species, that is, sustain one or more major functions within their ecosystem (Naeem et al., 2002). If phylogeny and functions are related, then conservation effort can be concentrated on the subset of “phylogenetic functionally” singular species. But in the case of a mismatch between phylogenetic and functional singularities, then conservation efforts would be greater as it has to target both phylogenetic and functionally singular species.

Our aim was to test whether phylogeny may be a proxy of functional ecology in a clade comprising several threatened species, that is, sharks. This goal is achieved by studying the relationships between phylogeny and functions and completed by several analytical steps. First, a comparison of phylogenetic and functional pairwise distances between species, and of phylogenetic and functional trees, is calculated. These first two analytical steps are addressed to figure out the importance of the phylogenetic tree reconstruction when assessing phylogenetic diversity as a proxy of functional diversity, a recent issue in ecological literature (Cadotte, 2015; Rangel et al., 2015). Second, the quantification of the phylogenetic signal on a quantitative estimation of species functional identity and the comparison of phylogenetic and functional singularities of species will allow to address evolutionary and conservative challenges.

## 2 METHODS

### 2.1 Extraction of DNA sequences and phylogenetic tree

**Figure 1:**
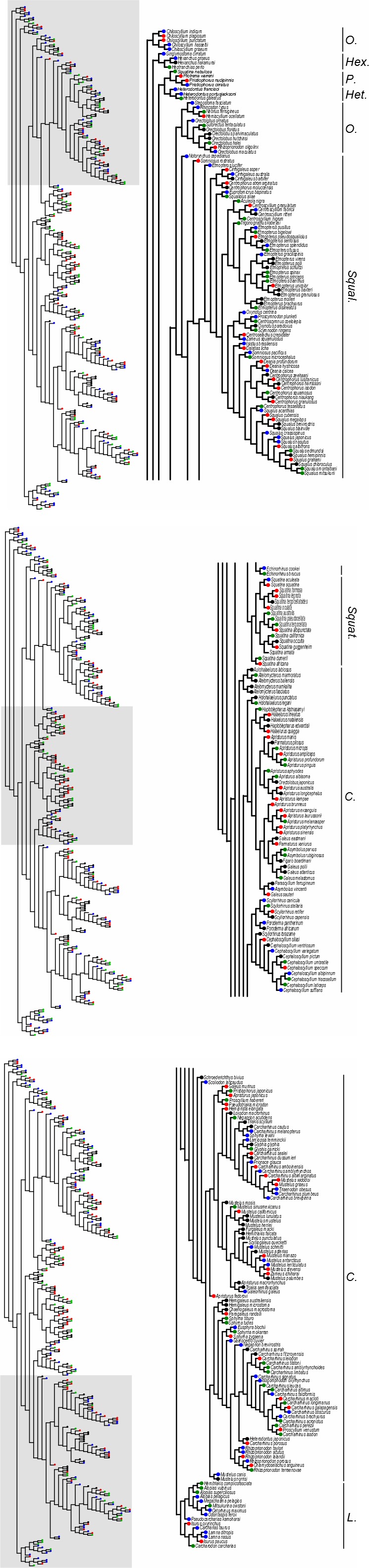
Phylogenetic tree of sharks. Tips colors represent the four quartile of the quantitative measure of functional singularity, from blue (low functional singularity) to yellow (high functional singularity)

Four commonly available genes were collected to assess phylogenetic relationships between sharks. Three mitochondrial DNA sequences (Cytochrome‐*b*, hereafter cyt‐*b*; 12S and 16S) as well as one nuclear gene, that is, the Recombination‐Activating Gene 1 (hereafter RAG1) were obtained from GenBank (Benson et al., 2013). We are aware that GenBank sequences are individual specimen based, and prone to misidentification of species (see Naylor et al., 2012), but they were primarily chosen to combine both mitochondrial and nuclear genes in a multi*loci* approach, in order to avoid single‐*locus* analysis bias (McCormack, Hird, Zellmer, Carstens, & Brumflied, 2012; Nichols, 2001). Then, for each gene, sequences were aligned using MAFFT (Katoh, Misawa, Kuma, & Miyata, 2002) and phylogenies were calculated with ClustalW (Larkin et al., 2007). Then, a super Tree was computed based on the four trees previously assessed. Super Tree is a multi*loci* approach that allows to output a single tree from a set of different trees with overlapping taxa (Liu, Yu, & Pearl, 2009; see also Appendix S1 for discussion on multi*loci* relevance for phylogenetic reconstruction). This technique transforms the topology of each tree into matrices, and combined and analyzed them with an optimization criterion, here Maximum Parsimony (Bininda‐Emonds, 2004; Bininda‐Emonds, Gittleman, & Steel, 2002). This final phylogenetic tree allowed to estimate phylogenetic relationships between 282 species representing the eight orders of sharks and 31 families (Figure 1 and Appendix S2).

### 2.2 Species traits and functional tree

Functional traits were collected from FishBase (Froese & Pauly, 2015) for the 282 species under study. These 13 traits were chosen to represent different life aspects of sharks, such as the habitat preference (range of usual depth, migration, usual habitat between shelf and slope and offshore and coastal areas, water preference,), the trophic ecology (position in the water column, trophic level, and maximum size). and behavior (activity time and schooling, see Table 1) following Poff (1997), Poff et al. (2006), and Stuart‐Smith et al. (2013). Correlation tests confirmed that they were not correlated (see Appendices S2 and S3 for functional traits database and correlations results between functional traits, respectively).

**Table 1:** Description of functional traits categorized into three trait groups and related function

Group	Traits	Related function
Habitat preference	Shallow/deep	Impacted ecosystem
	Migration	Impacted ecosystem
	Shelf/slope	Impacted ecosystem
	Offshore/coastal	Impacted ecosystem
	Water preference	Impacted ecosystem
Trophic ecology	Water column position	Feeding location
	Trophic level	Biomass and energy transfer
	Size max	Morphology
Behavior	Nocturnal	Activity time
	Schooling	Social behavior

Then, a functional tree was constructed as a hierarchical clustering calculated with the Gower distance and UPGMA on the 13 functional traits (Legendre & Legendre, 2012). The Gower distance is common to assess functional distances between species as it handles different types of data, such as functional traits, in the same analysis (qualitative, quantitative, binary etc., Petchey and Gaston, 2002a). UPGMA was chosen after the comparison of different clustering methods (single linkage, complete linkage, UPGMA, WPGMA, and Ward) by their correlation values between the cophenetic distance resulting from the hierarchical clustering and the initial distance between data (i.e., the best correlation implying a representative dendrogram of original distances (Mouchet et al., 2008). Here, Mantel test using 999 randomizations showed that the initial distance matrix and the cophenetic distance matrix from the functional tree were significantly correlated up to 77%. Despite this high correlation, we cannot exclude that the technique used to generate the dendrogram may have incidence on future results.

Considering the amount of “NA” (nonavailable data) in the functional database, a subdatabase excluding all “NA” (NA‐excluded) was used in later analytical steps to estimate how “NA” may affect results. Excluding “NA” decreased the number of species under study to 86. In the same line, the original functional trait database was also split into three functional subdatabases (habitat traits, trophic traits, and behavioral traits, Table 1) in order to estimate the impact of traits inclusion on analyses.

### 2.3 Statistical analyses

Different quantitative approaches were used to compare phylogeny and functions of sharks. These approaches were performed between phylogenetic data and functional data, this last being based on the original 13 functional traits database, and on the four subdatabases described before (NA‐excluded data, habitat traits, trophic traits, and behavioral traits). The first step was a comparison between the two distance matrices (pairwise cophenetic distances from the phylogenetic tree and pairwise Gower distances from functional traits) by a Mantel test (Mantel, 1967).

The second step was the direct comparison of phylogenetic and functional trees' topologies. Two metrics of difference between trees were computed: the topological difference (Penny and Hendy, 1985), based on the number of branches that differ between trees, and which ranged from 0 to 2*n*−6, *n* being the number of species. As we dealt with different number of species because of the NA‐excluded database (282 as opposed to 86), the relative topological difference (RTD) was calculated as the proportion of topological difference such as:

$$\text{RTD}=\frac{\text{topological difference}}{2n-6}$$

ranging from 0 (no difference) to 1 (completely different). The second metric was the branch length score (hereafter BLS, Kuhner & Felsenstein, 1994), which takes branch length into account (Steel and Penny, 1993). These two metrics were calculated on normalized trees, that is, with a total tree length equal to 1.

The third step was to calculate the phylogenetic signal on functional traits taken as a whole in a measure of “functional identity.” The “functional identity” was estimated by a Brownian simulation. This simulation allows to give a quantitative state for each tip of a tree (here species in the functional tree). As a consequence, the functional identity may be defined here as the estimation of the species place in the functional tree (see Revell, 2012, for further explanation). The phylogenetic signal of functional identity was calculated by the computation of both Moran's *I* and Abouheif's C_mean_. These two statistics estimate the deviation from the Brownian model of evolution for traits and were recently advised to measure phylogenetic signal (Münkemüller et al., 2012). Moran *I* and Abouheif's C_mean_ take values comprised between −1 (no phylogenetic signal) and 1 (complete phylogenetic signal).

The final step aimed to compare “species singularities.” The Evolutionary Distinctiveness index (ED, Isaac et al., 2007) was first calculated on the phylogenetic tree as a level of phylogenetic singularity (PS) for each species, and then calculated on the functional tree as species functional singularity (FS). This index is defined, for each branch, by its length divided by the number of species descendant from this branch. The singularity of a species is the sum of these values for all branches it descended from. To investigate the relationships between phylogenetic and functional singularities among species, a correlation of Pearson between species PS and species FS was computed. All analyses were conducted with packages “ape” (Paradis et al., 2004), “phytools” (Revell, 2012), “picante”(Kembel et al., 2010), and “vegan”(Oksanen et al., 2016) of the software R (R Core Team, 2015).

In order to support our present work, a supplementary phylogenetic tree based on a single sequence (cyt‐*b*) but computed with bootstrap procedures was also confronted to the four previously described analytical steps, and results were consistent with those presented in this study (see Appendices S4 and S5).

## 3 RESULTS

The phylogenetic tree (super Tree) of shark species showed an important diversification of sharks (Figure 1). Unsurprisingly, both squaliform and carcharhiniform represented the majority of species. Lamniform and squatiniform appeared closely related to carcharhiniform, while hexanchiform, heterodontiform, hexanchiform, orectobiliform, and pristiophoriform seemed to exhibit more complex phylogenetic relationships. However, values of functional singularity were clearly not related to each other along the tree (Figure 1).

The different analytical steps were all consistent. The first step consisted in the comparison of phylogenetic and functional distances by Mantel tests. The test was significant but particularly low between phylogenetic distances and functional distances calculated with the 13 functional traits (*r* = .067, *p*‐value <.05, Table 2), meaning that phylogenetic and functional pairwise distances between shark species were not correlated. When considering functional subdatabases, Mantel tests were also significant and particularly low between phylogenetic and functional distances calculated with habitat and trophic traits (*r* = .078 and 0.106, respectively, *p*‐values <.05, Table 2) and were not significant considering NA‐excluded and behavioral trait databases (*p*‐value >.05, Table 2).

**Table 2:** Results of Mantel tests between functional and phylogenetic pairwise distance matrices under different models based on the complete database (“Phylogeny ~ function”) and on several subdatabases (“NA‐excluded”, “habitat traits”, “trophic traits”, “behavioral traits”)

Model	Statistics	*p*‐value
Phylogeny ~ function	0.067	**<.05**
Phylogeny ~ NA‐excluded data	0.052	>.05
Phylogeny ~ habitat traits	0.078	**<.05**
Phylogeny ~ trophic traits	0.106	**<.05**
Phylogeny ~ behavioral traits	0.001	>.05

Values in bold are considered as significant (*p*‐value < 0.05).

**Figure 2:**
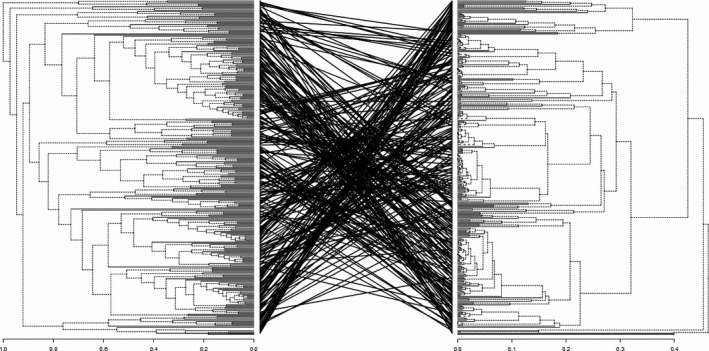
Tanglegram comparing the phylogenetic tree (left) and the functional tree (right) of sharks

The second step aimed to calculate two metrics comparing trees topologies. These metrics, measured between the phylogenetic tree and the functional tree based on the 13 functional traits, converged to a strong difference (Relative Topological Difference, RTD = 0.993; and Branch‐length Score, BLS = 2.247, Figure 2, Table 3). When these metrics were calculated with functional trees based on the four subdatabases (NA‐excluded, habitat traits, trophic traits, and behavioral traits), RTD ranged from 0.988 to 1, and BLS from 2.355 to 2.627 (Table 3), confirming the strong difference between the phylogenetic tree of sharks and their functional trees.

**Table 3:** Results of Relative Topological Difference (RTD) and branch length score (BLS) between functional and phylogenetic trees under different models based on the complete database (“Phylogeny ~ function”) and on several subdatabases (“NA‐excluded”, “habitat traits”, “trophic traits”, “behavioral traits”)

Model	RTD	BLS
Phylogeny ~ function	0.993	2.247
Phylogeny ~ NA‐excluded data	0.988	2.627
Phylogeny ~ habitat traits	1.000	2.355
Phylogeny ~ trophic traits	0.996	2.385
Phylogeny ~ behavioral traits	1.000	2.428

The third step used Moran's *I* and Abouheif's C_mean_ as estimations of the phylogenetic signal on species functional identity. When functional identities of sharks species were measured as their positions in the functional tree computed with the 13 functional traits, phylogenetic signal appeared relatively low (*I *=* *0.322, and C_mean_ = 0.336, Table 4) but significant (*p*‐values <.05). This pattern was also expressed for estimation of phylogenetic signal on functional identities calculated with three functional trees based on subdatabases (NA‐excluded, habitat traits, and trophic traits), *I* and C_mean_ being always significant (*p*‐values <.05) and ranging from 0.110 to 0.418 (Table 4). Both Moran's *I* and Abouheif's C_mean_ were not significant for behavioral traits (*p*‐values >.05, Table 4).

**Table 4:** Results of the estimation of phylogenetic signal on species functional identity calculated with Moran's *I* and Abouheif's C_mean_ under different models based on the complete database (“Phylogeny ~ function”) and on several subdatabases (“NA‐excluded”, “habitat traits”, “trophic traits”, “behavioral traits”)

Model	*I*	C_mean_	*p*‐value
Phylogeny ~ function	0.322	0.336	**<.05**
Phylogeny ~ NA‐excluded data	0.269	0.277	**<.05**
Phylogeny ~ habitat traits	0.110	0.120	**<.05**
Phylogeny ~ trophic traits	0.415	0.418	**<.05**
Phylogeny ~ behavioral traits	0.006	0.013	>.05

Values in bold are considered as significant (*p*‐value < 0.05).

**Figure 3:**
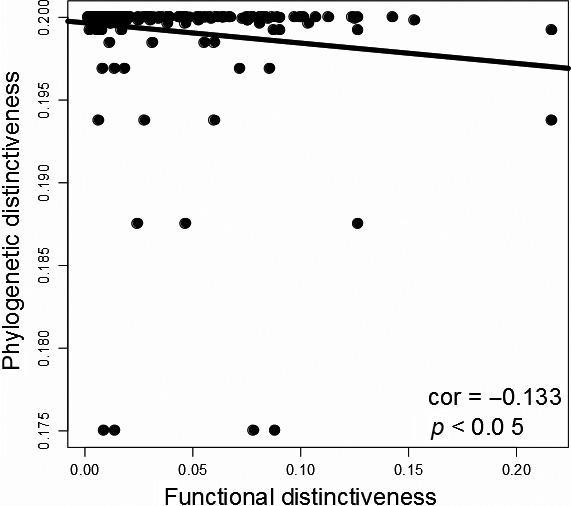
Correlation between phylogenetic singularity (PS,* y*‐axis) and functional singularity (FS,* x*‐axis) in shark species. Dots represent species (*n* = 282)

The final step was the correlation between species phylogenetic singularity (PS) and functional singularity (FS). When FS was calculated with the functional tree based on the 13 functional traits, species singularities were weakly correlated (Pearson's product moment correlation coefficient = −0.133, *p*‐value <.05, Table 5 and Figure 3), as for the correlation calculated between phylogenetic singularities and behavioral traits' functional singularities (Pearson's product moment correlation coefficient = −0.161, *p*‐value <.05, Table 5). Results were no longer significant for FS calculated with functional trees based on NA‐excluded data (correlation coefficient = 0.122, *p*‐value >.05), habitat traits (correlation coefficient = −0.041, *p*‐value >.05), and trophic traits (correlation coefficient = −0.044, *p*‐value >.05, Table 5).

**Table 5:** Results of Pearson's correlations (cor) between species phylogenetic (PS) and functional singularities (FS) under different models based on the complete database (“Phylogeny ~ function”) and on several subdatabases (“NA‐excluded”, “habitat traits”, “trophic traits”, “behavioral traits”)

Model	Cor	*p*‐value
Phylogeny ~ function	−0.133	**<.05**
Phylogeny ~ NA‐excluded data	0.122	>.05
Phylogeny ~ habitat traits	−0.041	>.05
Phylogeny ~ trophic traits	−0.044	>.05
Phylogeny ~ behavioral traits	−0.161	**<.05**

Values in bold are considered as significant (*p*‐value < 0.05).

## 4 DISCUSSION

The chosen clade‐based approach allowed to focus on a single evolutionary context in the study of the relationships between phylogeny and functions. As this relationship depends on the evolutionary history of the clade, it may be hard to generalize the present results to other taxa because evolutionary history is species dependant (Srivastava et al., 2012). In the case of sharks, the relationships between phylogeny and functions were either weak or not detected depending on the analytical approach.

One limitation in our study may be the reconstruction of the phylogenetic tree. Recent studies on shark species presented two approaches. One estimated phylogeny of a large number of species (595) using a single gene (NAPH2, Naylor et al., 2012), and the other implied less species but several genes (Sorenson et al., 2014; Vélez‐Zuazo and Agnarsson, 2011). Here, we decided to follow the second approach, as our aim was to explore whether phylogeny may be a tool to assess functional identity, not describing the evolutionary history of shark species. Another limitation would be the pool of functional traits, but they were chosen according to their availability and the function they represent, and were consistent with those used in other studies (Albouy et al., 2014; Cadotte et al., 2011; Poff, 1997; Stuart‐Smith et al., 2013). Furthermore, we tried to compensate this weakness by repeating analyses with subdatabases (NA‐excluded data, habitat traits, trophic traits, and behavioral traits). Results from these subdatabases were relatively consistent with those computed with all 13 functional traits, enhancing our principal results.

In this study, the comparison of phylogenetic and functional distances between species did not show a clear relationship, the Mantel statistics being comprised between 0.05 and 0.11, when significant. This preliminary analytical step tried to be independent from tree reconstruction, at least for the functional tree, as phylogenetic distances were calculated on the phylogenetic tree. Although methodological, this point have to be considered in biodiversity–ecosystem functioning (BEF) studies (Albouy et al., 2014; Guilhaumon et al., 2014; Mouchet et al., 2008). Several authors indeed recently pointed out that researchers need to be careful when they rely on phylogenetic tree reconstruction for ecological or biogeographical studies (Cadotte, 2015; Rangel et al., 2015). In our case study, the phylogenetic tree was reconstructed as a SuperTree, a multi*loci* approach, combined with Maximum Parsimony optimization. However, it is clear that other *loci* and related phylogenetic trees, computed with evolution models such as GTR, F81, K80, or JC, as usually performed in phylogenetic studies, may have produced different results. These usual analytical steps were, in fact, tested and their results, supporting those with the SuperTree, are available in Supplementary Information 4 and 5.

A second step was the topological comparison between phylogenetic and functional trees. These trees appeared to be highly different (Relative Topological Difference >99%, branch length score >2), consistently with our previous result. It indicated that phylogenetic and functional organizations of species within the taxa differ, whether considering branch length or not. Taken together, these results converged to an absence of relationships between phylogeny and functional identity in shark species.

The pattern of this relationship between phylogeny and functions was under several hypotheses. The first one, implying a clear and positive relationship, was niche conservatism (Ackerly, 2009; Losos, 2008; Münkemüller et al., 2015; Srivastava et al., 2012; Wiens et al., 2010). It is based on the idea that functions are conservative traits along evolution, and thus that phylogenetically close species should exhibit similar traits and be functionally close (Losos, 2008; Wiens et al., 2010). In that case, a phylogenetic signal would be detected on species traits. In our study, we quantified the phylogenetic signal on an estimation of the functional identity of shark species using Moran's *I* and Abouheif's C_mean_. A significant but weak phylogenetic signal was detected, implying that functional identities of species are more different than expected by their phylogenetic closeness. However, differences between functional traits used in this step can explained this pattern. When functional traits were separated (subdatabases), a particular pattern appeared: a relatively strong phylogenetic signal was detected for trophic traits, but not for behavioral traits nor for habitat traits. This may be explained by the fact that trophic traits included trophic level and maximum size, which are probably genetically coded and thus conserved along evolution. On the opposite, behavioral traits showed no phylogenetic signal, which was unsurprising as behavior is generally considered as a much more plastic trait than any other phenotypic aspects, like size or morphology (Dall, Bell, Bolnick, & Ratnieks, 2012; West‐Eberhard, 1989). This result highlights that it may be important, for further functional studies, to test different combinations of functional traits in order to better understand species' functions organization. To remind, our goal here was to test whether phylogeny may become a proxy for functional identity, this last comprising the complete role of a species within its ecosystem. Our main result was the apparent absence of functional niche conservatism among the shark taxa, considering all functional traits. This result is in line with several previous studies (see Losos, 2008), and thus supports the idea that conservatism may only occur for some traits but not for the whole functional niche (Pearman et al., 2008).

This weak phylogenetic signal may be explained by two others evolutionary histories related to the absence of phylogenetic signal on habitat traits: adaptive radiation and evolutionary convergence (Mouquet et al., 2012; Srivastava et al., 2012). First, adaptive radiation happens when a high rate of traits divergence, due to competitive exclusion and character displacement, produces ecologically different species despites their phylogenetic closeness (Dayan & Simberloff, 2005; Schluter, 2000, 1996). Second, evolutionary convergence implies that phylogenetically distant species, if they face relatively similar environment, may adopt similar traits, and thus became more ecologically similar than expected by phylogeny (Cadotte et al., 2013; MacArthur & Levins, 1967). As sharks are one of the oldest taxa in vertebrates (Compagno, Dando, & Fowler, 2005), both processes may have occurred along their long evolutionary history (450 million years). It is indeed not possible to assess which process (or both) led to the weak phylogenetic signal on the functional identities.

Our main aim was to test whether phylogeny may be a proxy of functional ecology in order to target conservation efforts on evolutionary and ecological keystone species. It is of major importance, in a context of a “Noah's Ark problem,” that is, the emergency to protect subset of species that may matter in a changing world, to wisely identify species being targeted by conservation measures. Sharks were chosen in particular because they include highly threatened species, as their life traits (slow growth, tardive sexual maturity, and low fecundity) make them particularly vulnerable (Compagno, 1990) to their currently high exploitation (Clarke et al., 2006). At the same time, they exhibit a high diversity of ecological traits and probably encompass specialist species. It may thus be expected that sharks assume a unique diversity of functions within many ecosystems (Ferretti, Worm, Britten, Heithaus, & Lotze, 2010). In this study, the comparison between phylogenetic and functional singularities of shark species was an important step to determine whether conservation targets may become common between two objectives. To remind, the first conservation objective focuses on genetic diversity, the second focusing on functions that matter for ecosystems. We found a weak or a nonsignificant correlation between species’ phylogenetic and functional singularities, depending on functional traits selected. The absence of a clear correlation between phylogenetic and functional identities means that conservation efforts should be concentrated on two subsets of shark species. First, on phylogenetically singular species to protect genetic diversity and thus adaptive potential facing the current changing environment (Isaac et al., 2007; Mace et al., 2003; Naeem, Duffy, & Zavaleta, 2012). Second, functionally unique species, in order to conserve singular functions within ecosystems to insure ecosystem functioning, goods, and service maintaining (Cadotte et al., 2011; Cadotte & Jonathan Davies, 2010; Naeem et al., 2012; Petchey and Gaston, 2002b).

The mismatch between phylogeny and functions was already reported in numerous studies that focused on communities’ structure rather than clade composition (see Losos, 2008). It may be explained by several factors such as (i) only some characters have a phylogenetic signal (trophic for example); (ii) along the colonization of their respective habitats, a convergent evolution constrained functional trait diversification; (iii) a blasted diversification of functional traits during past interaction with co‐occurring species, *via* character displacement for example (Losos, 2008), the latter being known as the “Evolutionary Interaction Hypothesis” (Prinzing et al., 2008). It is now clear that we need more studies to verify whether the absence of relationships between phylogeny and functions is common in other clades in order to help for wisely choosing conservation targets under a changing world.

## CONFLICT OF INTEREST

None declared.
