# Supplementary material for: Assessing the relationships between phylogenetic and functional singularities in sharks (Chondrichthyes)
Source: Ecol Evol. 2017 Jul 4;7(16):6292–303. doi: 10.1002/ece3.2871 (PMC5574805; doi:10.1002/ece3.2871)
Supplement: Supplementary file 1 [file ECE3-7-6292-s001.docx]

**Title: Assessing the relationships between phylogenetic and functional singularities in sharks (Chondrichthyes)**

Cachera Marie^1*^, Le Loc’h François^2^

^1^ SHOM, 13, rue du Chatellier, CS 92803, 29228 Brest cedex 2, France

^2^ UMR LEMAR CNRS/UBO/IRD/Ifremer, IUEM, Rue Dumont d’Urville, Technopôle Brest Iroise, 29280 Plouzané, France

* Corresponding author: [marie.cachera@gmail.com](mailto:marie.cachera@gmail.com)

**Tanglegram between mitochondrial DNA-based tree (on the left) and nuclear DNA-based tree (on the right) of shark species.** Mitochondrial DNA included Cytochrome-*b*, 12S and 16S sequences. Nuclear DNA included Recombination-Activating Gene 1 sequences.


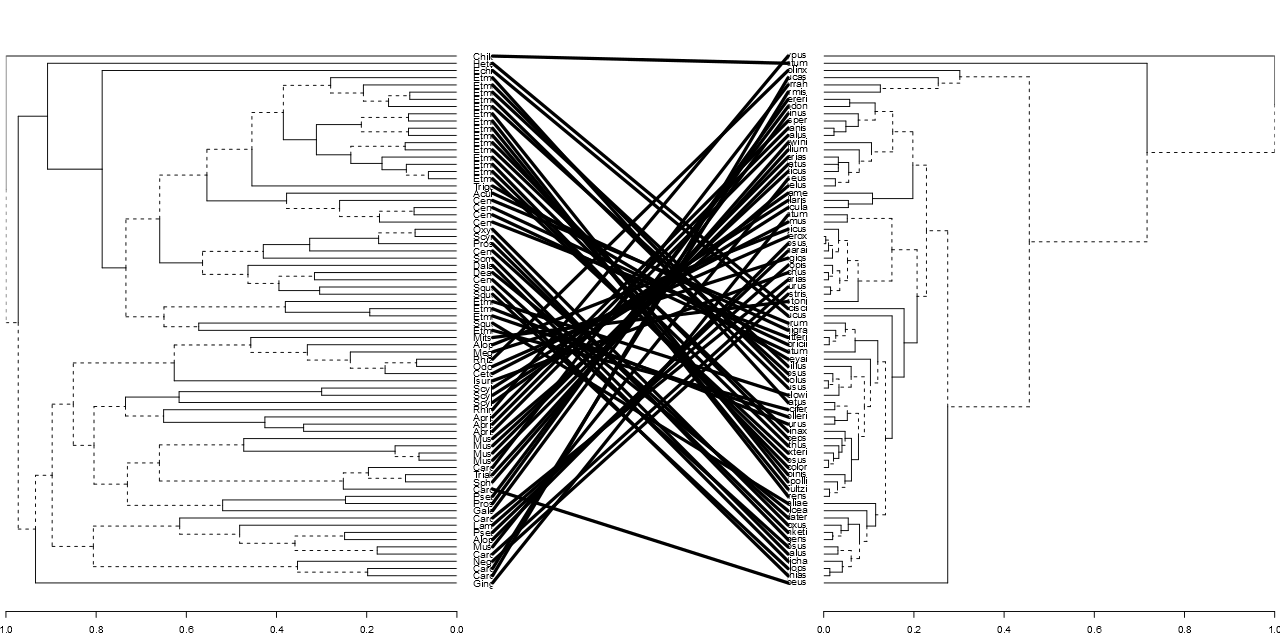


This tanglegram represents the comparison of two phylogenetic trees. The first tree on the left was based on 3 mitochondrial DNA sequences (cyt-*b*, 12S and 16S) and the second on 1 nuclear DNA sequence (Recombination-Activating Gene 1), with common shark species only. All 4 sequences were extracted from GenBank (Benson et al. 2013). To numerically complete this representation, the correlation between cophenetic distances was computed.

The tanglegram exhibited a clear difference between the two phylogenetic trees, in agreement with the significant correlation between their cophenetic distances (R² = 0.41, *P*-value < 0.05).

The mismatch between mitochondrial and nuclear gene trees was already studied and usually explained by (i) the difference in substitution rates between mitochondrial and nuclear DNA; and (ii) the fact that mitochondrial DNA reconstructs maternal lineage while nuclear DNA is biparentally inherited (Cummings et al. 1995). Historically, to assess phylogenetic relationships at species-level, a mitochondrial approach was generally preferred (using cyt-*b* in particular), but with the increasing availability of DNA sequences, it is now widely advised to use a combination of independent mitochondrial and nuclear sequences (Pamilo & Nei 1988; Moore 1995; Cummings et al. 1995), as in our study.

Benson DA, Cavanaugh M, Clark K, Karsch-Mizrachi I, Lipman DJ, Ostell J, Sayers EW. 2013. GenBank. Nucleic Acids Research **41**:D36–D42.

Cummings MP, Otto SP, Wakeley J. 1995. Sampling properties of DNA sequence data in phylogenetic analysis. Molecular Biology and Evolution **12**:814–822.

Moore WS. 1995. Inferring Phylogenies from mtDNA Variation: Mitochondrial-Gene Trees Versus Nuclear-Gene Trees. Evolution **49**:718–726.

Pamilo P, Nei M. 1988. Relationships between gene trees and species trees. Molecular Biology and Evolution **5**:568–583.
