# Supplementary material for: Assessing the relationships between phylogenetic and functional singularities in sharks (Chondrichthyes)
Source: Ecol Evol. 2017 Jul 4;7(16):6292–303. doi: 10.1002/ece3.2871 (PMC5574805; doi:10.1002/ece3.2871)
Supplement: Supplementary file 3 [file ECE3-7-6292-s003.docx]

**Title: Assessing the relationships between phylogenetic and functional singularities in sharks (Chondrichthyes)**

Cachera Marie^1*^, Le Loc’h François^2^

^1^ SHOM, 13, rue du Chatellier, CS 92803, 29228 Brest cedex 2, France

^2^ UMR LEMAR CNRS/UBO/IRD/Ifremer, IUEM, Rue Dumont d’Urville, Technopôle Brest Iroise, 29280 Plouzané, France

* Corresponding author: [marie.cachera@gmail.com](mailto:marie.cachera@gmail.com)

**Pairwise Kendall τ and corrected by Bonferroni *P*-values (in brackets) between binary functional traits of shark species.**

Nocturnal schooling shelf slope offshore coastal

Nocturnal 1.00

Schooling 0.03 (0.59) 1.00

Shelf 0.05 (0.55) 0.15 (0.08) 1.00

Slope -0.15 (0.08) 0.07 (0.41) 0.16 (0.06) 1.00

Offshore -0.06 (0.46) 0.03 (0.68) 0.11 (0.19) -0.22 (0.01) 1.00

Coastal -0.06 (0.49) 0.16 (0.06) -0.03 (0.72) -0.03 (0.76) 0.06 (1.00

**Correlation matrix of Chi2 between qualitative functional traits of shark species. *** = P-values < 0.001.**

Position Migration Preference

Position

Migration 81.898***

Preference 220.36*** 30.09***

**Pearson correlation between Size max and Trophic level of shark species:** Pearson moment = 0.061, *P*-value = 0.422.
