# Supplementary figures and images for: Assessing the relationships between phylogenetic and functional singularities in sharks (Chondrichthyes)
Source: Ecol Evol. 2017 Jul 4;7(16):6292–303. doi: 10.1002/ece3.2871 (PMC5574805; doi:10.1002/ece3.2871)

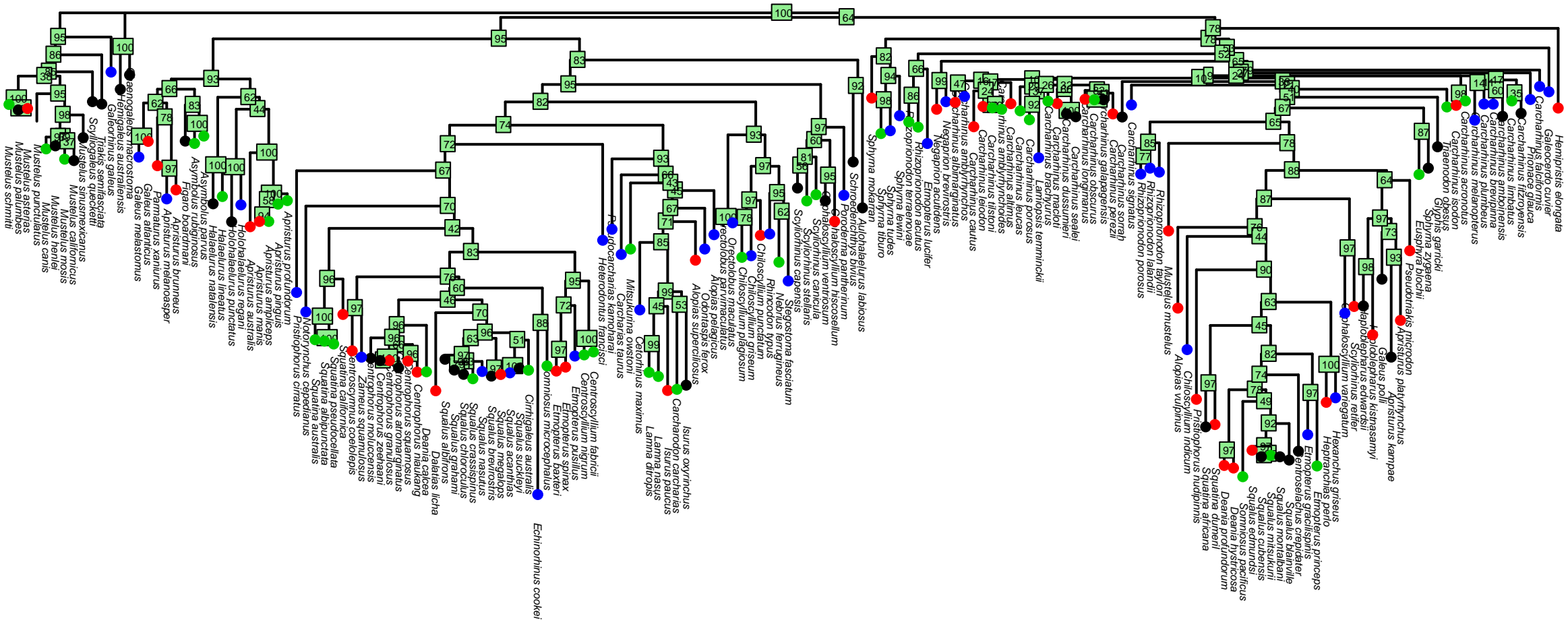

Supplement: Supplementary file 4 [file ECE3-7-6292-s004.pdf]
