# Supplementary material for: Assessing the relationships between phylogenetic and functional singularities in sharks (Chondrichthyes)
Source: Ecol Evol. 2017 Jul 4;7(16):6292–303. doi: 10.1002/ece3.2871 (PMC5574805; doi:10.1002/ece3.2871)
Supplement: Supplementary file 5 [file ECE3-7-6292-s005.docx]

**Title: Assessing the relationships between phylogenetic and functional singularities in sharks (Chondrichthyes)**

Cachera Marie^1*^, Le Loc’h François^2^

^1^ SHOM, 13, rue du Chatellier, CS 92803, 29228 Brest cedex 2, France

^2^ UMR LEMAR CNRS/UBO/IRD/Ifremer, IUEM, Rue Dumont d’Urville, Technopôle Brest Iroise, 29280 Plouzané, France

* Corresponding author: [marie.cachera@gmail.com](mailto:marie.cachera@gmail.com)

Methods

**Phylogenetic tree reconstruction**

A total of 168 shark species were common and available between cyt-*b* and functional traits databases. Cyt-*b* sequences were aligned using ClustalW (Larkin et al. 2007); and the best-fit maximum likelihood substitution model was selected and its parameters values were optimized (Paradis 2011). Here, optimized GTR model (Generalized Time Reversible, Lanave et al. 1984,) was chosen according to results of the following model test:

| Model | df | logLik | AIC | BIC |
| --- | --- | --- | --- | --- |
| JC | 333 | -53468.287 | 107602.575 | 109095.450 |
| F81 | 336 | -52540.876 | 105753.751 | 107260.075 |
| K80 | 334 | -51261.360 | 103190.720 | 104688.078 |
| HKY | 337 | -50608.195 | 101890.391 | 103401.198 |
| SYM | 338 | -49257.201 | 99190.402 | 100705.693 |
| **GTR** | **341** | **-49115.591** | **98913.182** | **100441.922** |

Then, a 100 non-parametric bootstrapped phylogenetic trees were produced in order to add a probability to every branch of the phylogenetic tree. All these preliminary step to determine the best model and the relevant phylogenetic tree were motivated by the most recent literature, advising the importance of selection the best model of evolution in phylogenetic-ecosystem functioning studies.

Results

The phylogenetic tree of the 168 shark species under study appeared relatively representative since the mean bootstrap values of nodes was 82% after 100 bootstrapping. The different analytical steps resulting from this primary phylogenetic tree were all consistent. The Mantel test between phylogenetic and functional pairwise distances was not significant (r = -0.008, *P*-value > 0.050), meaning that phylogenetic and functional pairwise distances between shark species were not correlated. The comparison of phylogenetic and functional trees topologies converged to an important difference (RTD = 0.970 and branch length score = 0.715). Moran’s *I* and Abouheif’s C_mean_, measuring the phylogenetic signal on the quantification of species functional identity, appeared low (*I* = 0.103, and C_mean_ = 0.105) but significant (*P*-values < 0.050). Finally, species phylogenetic singularity (PS) and functional singularity (FS) were not correlated (Pearson's product moment correlation coefficient = 0.116, *P*-value > 0.050).

These results were supported by their equivalent calculated with the 100 phylogenetic trees from bootstrapping procedures. Mantel r and *P*-value calculated with the primary phylogenetic tree were included within distributions of Mantel r and *P*-values calculated with the 100 bootstrapped phylogenetic trees supporting the non significance. The distribution of topological difference as well and the branch-length score also supported previous value. Results from Moran’s *I*, Abouheif C_mean_ and Pearson correlation tests between bootstrapped trees and the functional tree clearly did support previous analyses based on the primary phylogenetic tree.

**Hereafter results of analytical steps on the relationships between phylogeny and function with primary phylogenetic tree and its 100 bootstrapped.** Value of Mantel r between phylogenetic and functional distances and related *P*-values. Values of topological differences between phylogenetic and functional trees and values of the branch-length scores between phylogenetic and functional trees. Moran *I* of phylogenetic signal on functional identity and related *P*-values. Abouheif’s C_mean_ of phylogenetic signal on functional identity and related *P*-values. Pearson correlation coefficients between phylogenetic and functional singularities and related *P*-values. The red dot is the value calculated with the phylogenetic tree of the study. *P*-values under 0.05 were considered significant.


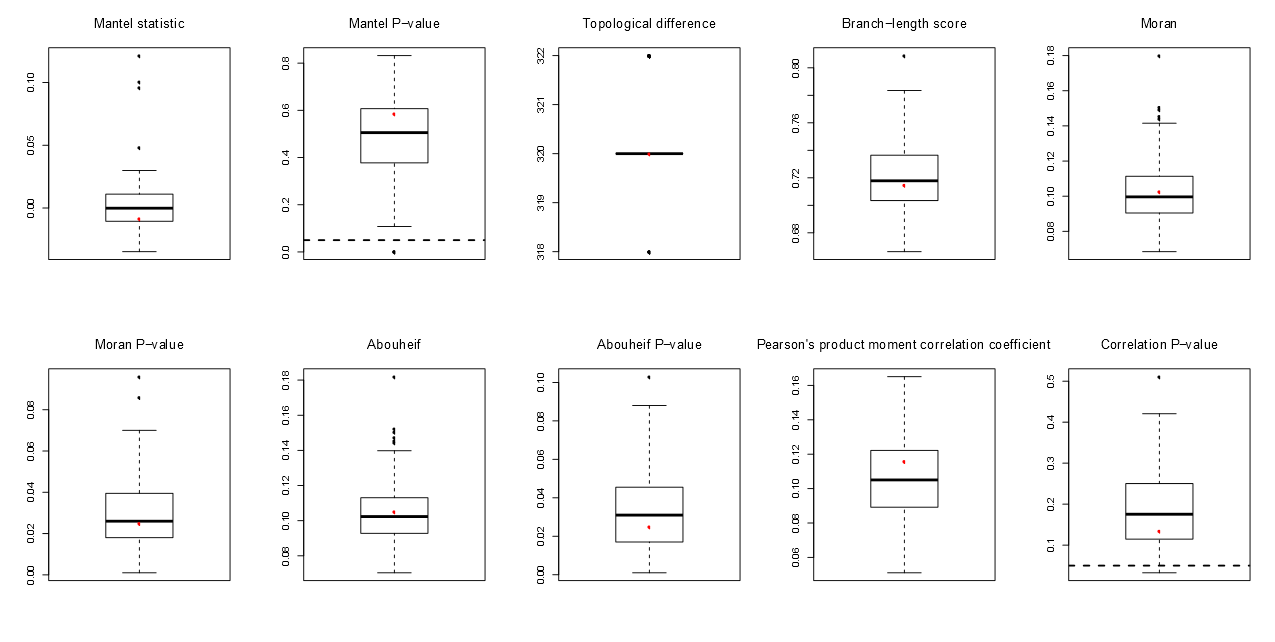


Lanave C, Preparata G, Saccone C, Serio G. 1984. A new method for calculating evolutionary substitution rates. Journal of Molecular Evolution **20**:86–93.

Larkin MA et al. 2007. Clustal W and Clustal X version 2.0. Bioinformatics **23**:2947–2948.

Paradis E. 2011. Analysis of Phylogenetics and Evolution with R. Springer Science & Business Media.
